# Supplementary material for: MetaRibo-Seq measures translation in microbiomes
Source: Nat Commun. 2020 Jun 29;11:3268. doi: 10.1038/s41467-020-17081-z (PMC7324362; doi:10.1038/s41467-020-17081-z)
Supplement: Supplementary file 10 — Supplementary Data 7 [file 41467_2020_17081_MOESM10_ESM.zip › File2/Confidence_High_Taxonomy/349561_out.krona.html]

Javascript must be enabled to view this page.

members
magnitude
magnitudeUnassigned
count
unassigned
taxon
rank

349561\_out

3

3
superkingdom
2

phylum
1239
3

3
class
186801

3
186802
order

3
186803
family

genus
841
3


SRS019068\_contig\_number\_76671SRS064276\_contig\_number\_40163SRS144135\_contig\_number\_21532
3
360807
species
